# Extracting the First Permanent Molars

**Published:** 1902-10-15

**Authors:** H. A. Pullen

**Affiliations:** Buffalo, N. Y.


### EXTRACTING THE FIRST PERMANENT MOLARS.

BY DR. H. A. PULLEN, BUFFALO, N. Y.

Before discussing in detail the indications for extraction of the first permanent molars, I wish to mention some of the points concerning these teeth which prove them to be the most important of the permanent teeth to preserve, at the time usually advocated for their extraction.

First. They are the first of the permanent teeth to erupt, and during the period of removal of the temporary dentition, afford the broadest and best masticating surface in the mouth.

Second. By reason of their great strength and size, these are the only teeth that can serve as a means of preserving the normal relationship between the jaws, and consequently the symmetry of the face at a time when no others of the permanent set, except the incisors, are erupted to occlusion.

Third. Their presence is an aid in securing developmental pressure in the development forward of the jaws.

Fourth. Statistics comparing the relative frequency of caries between the first and second molars prove the second molar more frequently carious than the first.

Fifth. The first molar, on the average, is a better constructed tooth than either the second or the third molar.

Sixth. Its extraction is the cause of more cases of irregularity than is generally supposed.

In my opinion, from the examination of a great many cases in practice, I believe the extraction of this tooth to be the cause of a very large percentage of irregular teeth. It is utter folly to believe that the symmetrical extraction of these teeth before the eruption of the second molars will give more room in the jaws for the teeth "to be erupted anteriorly and posteriorly." The jaw is smaller and the number of teeth is less than it otherwise would have been. Irregular teeth do not straighten themselves simply because the first molars are extracted. Extraction of the first molars will many times shorten the bite, allowing the jaws to come closer together, the bicuspids not being allowed to erupt fully, the undue pressure on the upper anterior teeth causing their protrusion and the lower to come in contact with the gums. Even when symmetrical extraction of the first molars has been performed with the view of their displacement by the second molars, it happens more often than otherwise that the second molars tilt forward into space, and not only form lodging places for food, but cause some serious malocclusion.

To relieve suffering, it seems to me, would be an excuse for the extraction in a majority of these cases presenting, for they belong to the class of patients who neglect the deciduous teeth and first molars until the pain of the toothache drives them to the dentist.

There are chances of these patients suffering more in after years from the extraction of these teeth occasioning faulty occlusion and impaired mastication which it would be impossible to relieve. Thus the extraction for the relief of the suffering would be but temporary. I believe that we can get around this difficulty with proper treatment.

It seems to me that if we let indifference upon the part of the patient as regards the saving of a first molar affect our judgment or deter us from doing the right thing in the case, even though we lose a dollar or two, we are not holding up a standard which is going to benefit our patients or our profession. It is our business to advise correctly if we can, and if patients do not choose to abide by it, then let them go elsewhere where their every wish may be gratified. The future of our profession depends upon the conservation of the dental organs, and a small temporary pecuniary loss for giving the proper advice on the subject of extraction will weigh but little compared with what the profession will gain in the long run by our holding up the standard.

I do not mean to be understood as saying that the first molars should never be extracted under any conditions. If these teeth are in such a condition that they can not be treated, especially if they are destroyed so early in their career that the roots have not become fully developed, extraction is indicated. Sometimes, however, it may be possible to treat a first molar whose roots are not fully developed, and we may find it advisable to do so, even if they last only a few years, if we find that the preservation will prevent an irregularity. However, if the extraction of one or more first molars becomes a necessity, unless the second molars are erupting and the conditions are favorable for a continuity of the relationship between the jaws, I believe that we should take measures for preserving the proper relationship until the second molars erupt to occlusion.

If the second molars are already erupted, when extraction of the first molars is inevitable the space should be retained or filled with an artificial substitute to prevent serious malocclusion. Of course the almighty dollar is a factor in these cases, and each of us knows how much time we are willing to give away in treating and preserving pulpless first molars. For myself I think we gain in the long run by giving the best possible service in every case, whether to rich or poor; the knowledge of having done the right thing is a soothing balm to the conscience.

As regards symmetrical extraction of the first molars I must say that I am not a believer in its so-called beneficial results. If the advocates of this method placed as much value on the individual tooth as I do, I am sure their attempts at improving on nature would very shortly stop.

The case of the thumb-sucker is cited as being a case where extraction was indicated in the upper arch, but not in the lower. This statement I can not let go unchallenged, as we often find the relations between the jaws of the thumb-sucker normal mesio-distally, which in consequence is no indication whatever for extraction, the only treatment necessary to restoring normal occlusion and harmony of the occlusal planes being a slight operation on the anterior teeth immediately concerned with the habit.

Two distinct reasons or indications are given for such extraction, viz, to correct irregularity and to relieve suffering. The two reasons are such as might be given for the extraction of any other tooth in the arch. Still, I feel very much like even more restricting the indications for removing such an important tooth. Very rarely, indeed, if ever, have I been persuaded to extract this molar to correct an irregularity. I endeavor to maintain in my practice an unwritten law that it is only after the disease and the operator have had their struggle to a finish, with the disease still unconquered, that this tooth is handed over to the forceps for extraction.

The first permanent molars take their places in the arch at an important period in the physical development of the boy or girl, at a time when the being is full of life and activity, and consequently in the need of nourishing and properly masticated food. The temporary molars are now, in many cases, gradually breaking up and becoming of little use in masticating, so that the first molar is more intended by nature to assume a responsible position, not only as the pillar of mastication, but also to preserve the proper contour and character of the face by keeping the jaws sufficiently apart till the second molars come to the rescue. So when these molars are removed, say at the age of seven or eight, the patient has not only to contend for some years against defective mastication and a consequent impaired digestion, but there is also frequently noticeable a lack of character in the general make-up of the face.

Again, if they are not removed till after the eruption of the second molars, we have as a result the malposed or tilted tooth on each side of the space—a condition of affairs which every experienced and conscientious operator looks upon with regret.

There is no doubt, is these molars have to be removed, that the time for the operation is a few months previous to the eruption of the second molars. They will then have given several years of valuable service in masticating the food and building up the contour of the face. Also the second molars will before erupting have an opportunity of traveling forward and at least partially filling the space of the departed life. However, I have but one word of advice as to the extraction of these important teeth, and that is: Don't.

I have taken the liberty of getting the opinions of two or three prominent members of the profession on this subject, which with your permission I will quote:

Dr. Ottolengui, of New York, writes me in this way: "It is a very rare thing for me to extract permanent first molars. If the teeth have been badly neglected, so that early in life they become pulpless, and especially if they are already abscessed, I should consider it best to remove them. I should almost never remove one in which the pulp could be preserved alive, and have never done so for purposes of regulating."

Dr. J. Taft, Ann Arbor, gives his opinion as follows: "Permit me to say that I regard it as a disaster for one to lose the permanent first molar tooth. Such a breach can never be wholly repaired, either by transplanting or by the insertion of an artificial tooth. The removal of this tooth in every case, in a large degree disturbs the masticating ability of the denture."

Dr. C. N. Johnston, Chicago, speaks very emphatically on the subject. He says: "I should endeavor to save the permanent first molar even if I had to crown it early in life, and above all things I should aim to keep it sufficiently built up, either by crowning or filling, so that it would hold the jaws apart and add character to the face."

Dr. Peirce: "Before the permanent first molars are extracted there should be some assurance that there are third molars to erupt."—Discussion in *Cosmos*.

